# High SARS-CoV-2 viral load is associated with a worse clinical outcome of COVID-19 disease

**DOI:** 10.1099/acmi.0.000259

**Published:** 2021-09-21

**Authors:** María Eugenia Soria, Marta Cortón, Brenda Martínez-González, Rebeca Lobo-Vega, Lucía Vázquez-Sirvent, Rosario López-Rodríguez, Berta Almoguera, Ignacio Mahillo, Pablo Mínguez, Antonio Herrero, Juan Carlos Taracido, Alicia Macías-Valcayo, Jaime Esteban, Ricardo Fernandez-Roblas, Ignacio Gadea, Javier Ruíz-Hornillos, Carmen Ayuso, Celia Perales

**Affiliations:** ^1^​ Department of Clinical Microbiology, Instituto de Investigación Sanitaria-Fundación Jiménez Díaz University Hospital, Universidad Autónoma de Madrid (IIS-FJD, UAM), Av. Reyes Católicos 2, 28040 Madrid, Spain; ^2^​ Centro de Biología Molecular “Severo Ochoa” (CSIC-UAM), Consejo Superior de Investigaciones Científicas (CSIC), Campus de Cantoblanco, 28049, Madrid, Spain; ^3^​ Department of Genetics & Genomics, Instituto de Investigación Sanitaria-Fundación Jiménez Díaz University Hospital, Universidad Autónoma de Madrid (IIS-FJD, UAM), Av. Reyes Católicos 2, 28040 Madrid, Spain; ^4^​ Centre for Biomedical Network Research on Rare Diseases (CIBERER), Instituto de Salud Carlos III, 28029, Madrid, Spain; ^5^​ Department of Statistics, Instituto de Investigación Sanitaria-Fundación Jiménez Díaz University Hospital, Universidad Autónoma de Madrid (IIS-FJD, UAM), Av. Reyes Católicos 2, 28040 Madrid, Spain; ^6^​ Data Analysis Department, Instituto de Investigación Sanitaria-Fundación Jiménez Díaz University Hospital, Universidad Autónoma de Madrid (IIS-FJD, UAM), Av. Reyes Católicos 2, 28040 Madrid, Spain; ^7^​ Allergy Unit, Hospital Infanta Elena, Valdemoro, Madrid, Spain; ^8^​ Instituto de Investigación Sanitaria-Fundación Jiménez Díaz University Hospital, Universidad Autónoma de Madrid (IIS-FJD, UAM), Av. Reyes Católicos 2, 28040 Madrid, Spain; ^9^​ Faculty of Medicine, Universidad Francisco de Vitoria, Madrid, Spain; ^10^​ Centro de Investigación Biomédica en Red de Enfermedades Hepáticas y Digestivas (CIBERehd), Instituto de Salud Carlos III, 28029, Madrid, Spain

**Keywords:** COVID-19, risk factors, viral load

## Abstract

COVID-19 severity and progression are determined by several host and virological factors that may influence the final outcome of SARS-CoV-2-infected patients. The objective of this work was to determine a possible association between viral load, obtained from nasopharyngeal swabs, and the severity of the infection in a cohort of 448 SARS-CoV-2-infected patients from a hospital in Madrid during the first outbreak of the pandemic in Spain. To perform this, we clinically classified patients as mild, moderate and severe COVID-19 according to a number of clinical parameters such as hospitalization requirement, need of oxygen therapy, admission to intensive care units and/or death. Also, Ct values were determined using SARS-CoV-2-specific oligonucleotides directed to ORF1ab. Here we report a statistically significant association between viral load and disease severity, a high viral load being associated with worse clinical prognosis, independently of several previously identified risk factors such as age, sex, hypertension, cardiovascular disease, diabetes, obesity and lung disease (asthma and chronic obstructive pulmonary disease). The data presented here reinforce viral load as a potential biomarker for predicting disease severity in SARS-CoV-2-infected patients. It is also an important parameter in viral evolution since it relates to the numbers and types of variant genomes present in a viral population, a potential determinant of disease progression.

## Impact Statement

Viral load is one of the first parameters to be measured in viral infections since it reflects the amount of virions circulating in the infected individual. Furthermore, the greater the number of virions, the greater the number of different variants present in the sample. In particular, in the new SARS-CoV-2 pandemic, there is a continuous search and urgent need for factors that could improve early prognosis of COVID-19 disease progression. Although not all studies have correlated high viral load levels in infected individuals with more severe stages of COVID-19, in our cohort we found a clear association between them. SARS-CoV-2 from nasopharyngeal swabs is the first viral sample readily available from an infected patient. Our results indicate that, in addition to their value for PCR-based diagnosis of the infection, their viral RNA load is a valid prognostic parameter for disease progression. Patients with high nasopharyngeal viral loads should be prioritized for administration of suppressive antiviral treatments.

## Introduction

Coronavirus SARS-CoV-2 emerged in the human population in 2019 and is the causal agent of the new pandemic disease COVID-19 [1]. The virus has spread rapidly worldwide, and at the time of this writing there have been 129 215 179 confirmed COVID-19 cases, and 2 820 098 deaths worldwide, according to the WHO (https://covid19.who.int/); these numbers are increasing daily. Evolution of a virus in a specific host is defined by a number of closely related parameters, such as viral load, replication rate, genetic heterogeneity and viral fitness, that may influence virus adaptability, viral pathogenesis and disease progression [2, 3]. The replicative capacity of a virus is clinically relevant because it largely determines the viral load in infected individuals, and viral load influences disease manifestations [4].

In the case of SARS-CoV-2, it is a current matter of debate whether SARS-CoV-2 viral biomarkers, such as the diagnostic viral load, are able to predict progression of COVID-19 disease. A positive correlation was reported in a cohort of SARS-CoV-2-infected patients from China, showing that the viral load detected in the respiratory tract was positively linked to severity of lung disease [5]. In a related study, analysis of the viral RNA level in upper respiratory tract samples from 76 patients with COVID-19 revealed significantly lower Ct values (cycle threshold, which is inversely correlated with viral RNA level), and longer virus-shedding periods in those patients classified as severe, as compared with those who exhibited mild disease [6]. Additionally, a prospective study in a large hospitalized cohort of 1145 infected patients documented a significantly lower probability of survival in patients with high viral load than in those with low viral load [7]. Additional studies have since been published supporting the association between viral load and disease severity ([8–15], see also [16] for a review), whereas in other studies this correlation was not clear [17–22]. For example, Argyropoulos *et al*. documented that the diagnostic viral load level was lower in hospitalized than in non-hospitalized patients, resulting in a lack of correlation of viral load with admission to the intensive care unit (ICU), duration of oxygen support and overall patient survival [22]. Thus, the dynamics of viral load and its connection with different clinical parameters still need further characterization with additional large cohorts worldwide to define the possible association and the predictive value of viral load regarding disease progression and mortality. Our results indicate that diagnostic Ct values analysed from nasopharyngeal swab samples can be added to other predictive parameters to complete an early risk stratification of COVID-19 patients [23].

## Methods

### Patient cohort and stratification

Data collected included patient demographics, risk factors for SARS-CoV-2 disease and clinical information related to the time of SARS-CoV-2 diagnosis (Table 1). Patients were classified according to the following COVID-19-associated parameters: (1) need of hospital admission, (2) need for mechanical ventilation, (3) admission to the ICU and (4) death attributed to COVID-19. Patients were classified as mild, moderate and severe cases according to the requirement and the type of hospitalization: (1) mild symptoms (neither hospital admission nor ICU) (*n*=110), (2) moderate symptoms (hospitalization without ICU) (*n*=236) and (3) severe symptoms (hospitalization with admission to the ICU, and/or death) (*n*=102). Exceptions to these criteria are detailed in Table 1. Notably, the clinical relevance was defined before the data analysis was performed.

**Table 1.** Demographic data and pre-existing comorbidities in 448 SARS-CoV-2-infected patients classified by disease severity of COVID-19

Characteristic	Total (*n*=448)	Disease severity				
		Death/severe (*n*=102)	Moderate (*n*=236)	Mild (*n*=110)	*P*-value*	Significance*
Age (mean±sd, years)	71.04±18.29	79.92±15.33	73.25±16.59	58.05±17.50	1.49×10^−12^	***
Age >60 years (%)	321 (71.6 %)	91 (89.2 %)	185 (78.4 %)	45 (41.0 %)	1.11×10^−8^	***
Male (%)	205 (45.7 %)	49 (48.0 %)	122 (51.7 %)	34 (31.0 %)	1.40×10^−12^	***
Female (%)	243 (54.2 %)	53 (52.0 %)	114 (48.3 %)	76 (69.1 %)		
Hypertension (%)	236 (52.7 %)	69 (67.6 %)	134 (56.8 %)	33 (30.0 %)	1.14×10^−12^	***
Cardiac disease (%)	142 (31.7 %)	42 (41.2 %)	73 (30.9 %)	27 (24.5 %)	1.35×10^−12^	***
Diabetes (%)	8 (1.8 %)	2 (2.0 %)	5 (2.1 %)	1 (0.9 %)	1.08×10^−12^	***
Obesity (%)	17 (3.8 %)	5 (4.9 %)	8 (3.4 %)	4 (3.6 %)	1.37×10^−12^	***
Asthma (%)	29 (6.5 %)	8 (7.8 %)	16 (6.8 %)	5 (4.5 %)	1.42×10^−12^	***
COPD (%)	15 (3.3 %)	4 (3.9 %)	8 (3.4 %)	3 (2.7 %)	1.34×10^−12^	***
Hospitalization (%)†	365 (81.5 %)	102 (100 %)	236 (100 %)	27 (24.5 %)	1.21×10^−12^	***
ICU admission (%)‡	36 (8.0 %)	33 (32.3 %)	2 (0.8 %)	1 (0.9 %)	1.16×10^−12^	***
Conventional oxygen therapy (%)	288 (64.3 %)	102 (100 %)	183 (77.5 %)	3 (2.7 %)	1.10×10^−12^	***
Invasive mechanical ventilation (%)	19 (4.2 %)	19 (18.6 %)	0 (0 %)	0 (0 %)	1.25×10^−12^	***
Non-invasive mechanical ventilation (%)§	21 (4.7 %)	20 (19.6 %)	1 (0.4 %)	0 (0 %)	1.27×10^−12^	***
High-flow nasal cannulas (%)	19 (4.2 %)	19 (18.6 %)	0 (0 %)	0 (0 %)	1.43×10^−12^	***
Survival 90 days after diagnosis (%)||	366 (81.7 %)	27 (26.5 %)	233 (98.7 %)	106 (96.4 %)	8.44×10^−13^	***
Days since symptom onset¶	7.84±6.40	7.57±6.62	8.26±6.53	3.25±0.50	2.81×10^−12^	***

*P-values indicate correlation between viral load and disease severity after adjusting for the baseline characteristics, pre-existing comorbilities, hospitalization, oxygen therapy and mortality. Statistical significance: ***P <0.001; ANCOVA test.

†Exceptions are the following: the 27 patients classified as mild COVID-19 were hospitalized for causes other than SARS-CoV-2 infection. In the group moderate COVID-19, one patient was hospitalized for causes other than SARS-CoV-2 infection, but was assigned to this group due to COVID-19 pneumonia according to the clinical history.

‡Exceptions are the following: two patients in the group moderate COVID-19 and one patient in the group mild COVID-19 were admitted to the ICU for causes other than SARS-CoV-2 infection.

§Exceptions are the following: in the group moderate COVID-19, one patient required non-invasive mechanical ventilation during hospitalization due to a previous pathology.

||Exceptions are the following: three patients in the group moderate COVID-19 and four patients in the group mild COVID-19 died during the 90 days after diagnosis due to pathologies prior to SARS-CoV-2 infection.

¶Data available for 102 patients.

### Molecular testing for SARS-CoV-2 by Ct value measurements

Nasopharyngeal swabs were collected at Fundación Jiménez Díaz University Hospital by trained medical personnel from all patients included in the study due to suspected and then confirmed COVID-19 infection. After collection, the nasopharyngeal samples were transferred to viral transport media and transported to the Microbiology Department for molecular testing. RT-PCR to obtain diagnostic SARS-CoV-2 viral load was performed using VIASURE Real Time PCR Detection Kits by CerTest BIOTEC following the manufacturer's instructions. Ct values were calculated using SARS-CoV-2-specific oligonucleotides directed to ORF1ab.

### Statistics

A normality test for the Ct values was performed with the Lilliefors test (Kolmogorov–Smirnov) using software R version 4.0.2. The statistical significance of differences among viral load values according to infection severity was calculated by ANOVA and *t*-test with Bonferroni correction using GraphPad Prism 7.00. The association between viral load and disease severity adjusted by risk factors was calculated with analysis of covariance (ANCOVA) using software R version 4.0.2.

## Results

**Fig. 1.**
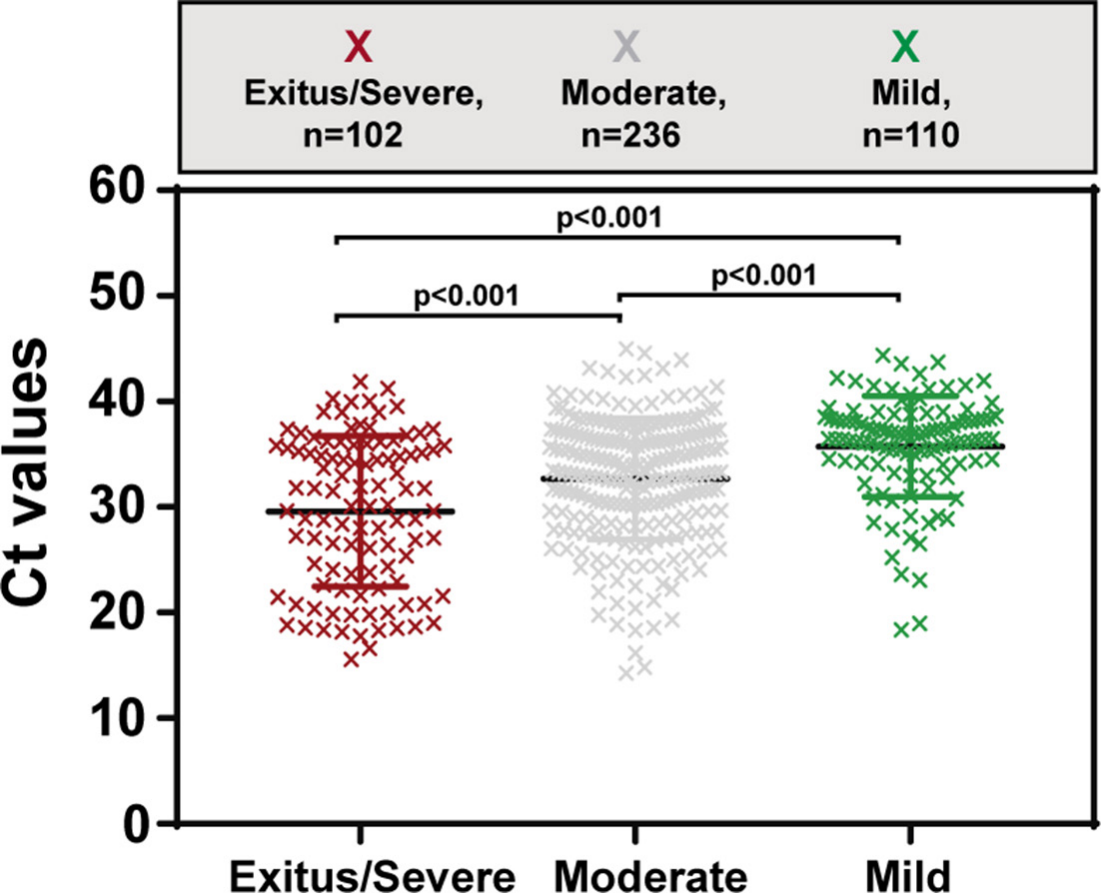
Correlation between viral load [measured by the Ct (number of PCR amplification cycles needed to cross the threshold detection level) value] and the severity of COVID-19 disease.

Given the disparate results of correlation between viral load and COVID-19 disease progression, we addressed this question with a large cohort of 448 patients admitted to the Fundación Jiménez Díaz Hospital (FJD, Madrid, Spain) from 3 to 29 April 2020 coinciding with the first COVID-19 outbreak in Spain. At the time of admission, all patients had clinical symptoms, and were confirmed to be positive for SARS-CoV-2 by a specific real-time RT-PCR (VIASURE Real Time PCR). The Ct values analysed correspond to the first available COVID-19 PCR-positive sample for each patient. The data follow a normal distribution [*P*-value=0.9246; Lilliefors (Kolmogorov–Smirnov test)]. Details regarding the clinical classification are described in the Materials and Methods. Mean Ct values for mild, moderate and severe COVID-19 patients were 35.75±0.45, 32.69±0.37 and 29.58±0.70, respectively. Univariate analysis showed statistically significant differences among viral load values according to infection severity (*P*<0.0001; ANOVA test) (Fig. 1). Specifically, average Ct values were significantly lower in the severe group as compared with the moderate and the mild disease groups, and also for the comparison between moderate and mild clinical categories (*P*<0.001 in all cases) (*t*-test with Bonferroni correction). Thus, Ct values in SARS-CoV-2-infected patients correlated positively with disease progression and poor prognosis.

Regarding host factors, it has been established that age greater than 65 years is a risk factor for developing acute respiratory distress syndrome (ARDS), a major complication of COVID-19 pneumonia, and that the risk of death increases with advanced age [24]. As a second factor, disease severity and mortality for males is significantly higher than for females, older men being the population most at risk [25, 26]. Additionally, several co-morbidities have been potentially associated with poor outcome, including hypertension (high blood pressure), cardiovascular disease, diabetes, obesity, and lung disease (asthma, excess post-exercise oxygen consumption) [27]. We have included these risk factor data in our patient cohort to assess their alignment with our disease severity–viral RNA load association. Interestingly, a significant association between viral load and infection severity was still observed after adjusting for age, sex, hypertension, cardiovascular disease, diabetes, obesity, asthma and chronic obstructive pulmonary disease (COPD) (see ANCOVA tests in Table 1). The significant difference in viral load between the three groups was not attributable to the percentage of hospitalization, the percentage of ICU admission or the percentage of the different types of oxygen therapy (see ANCOVA tests in Table 1).

## Discussion

Our results with a SARS-CoV-2 population, from a cohort that is different from those in previous studies on the influence of viral load on disease, suggest a positive association between viral load and COVID-19 disease severity. This conclusion is in agreement with many studies to date [5–10, 12–15, 28, 29] (see also [16] for a review), but in contrast, others did not show such an association [17–22]. A possibility to explain such disparate conclusions is the multiple differential criteria considered among studies, such as the sample size, the genomic region analysed, the clinical severity of the patients to be compared, or the standards to classify patients in clinical categories, among other features. Our findings are in agreement with other previously published studies showing a positive correlation between viral load and COVID-19 disease. For example, a longer persistence of high viral load viruses in respiratory samples of patients with severe disease than those with mild disease has been reported, suggesting that viral load may be a prognostic parameter [30, 31]. Ct values on admission were also predictors of COVID-19 severity in a small Japanese cohort of ICU patients (*n*=19) whose clinical classification as ‘critical’ and ‘non-critical’ illness was based on their oxygen demand during hospitalization [28]. In a large cohort of 4254 patients in a New York City medical centre, inpatients had significantly higher Ct values than outpatients, and lower Ct values at admission were significantly associated with mortality [29]. In a patient cohort in Italy, SARS-CoV-2 Ct at diagnosis, within the first week of disease onset, was associated with COVID-19-related death, disease severity, number of signs and symptoms, and for the first time the presence of 6 month sequelae [15].

The time elapsed between COVID-19 onset and the diagnostic swab collection may be an important variable to consider. However, only a few studies have reported this time interval [8, 12, 15]. Our cohort includes individuals whose swab is represented by the diagnostic one, and we recorded this time interval for 102 patients. On average nasopharyngeal swabs were obtained at 7.84±6.40 days after the onset of symptoms, which is within the time interval of active infection. It has been described that the highest SARS-CoV-2 viral load in throat swabs – and consequently the highest transmissibility peaks – is around 5–6 days after symptom onset [32], but this is an average, and values vary considerably among patients; the transmissibility window extends from a few days before symptom onset to 30 days in patients with severe disease [30, 33].

A correlation between high viral load and disease severity has been also found in other viral infections. Among children naturally infected with respiratory syncytial virus (RSV), increased viral load was associated with clinical severity of disease defined as an increased risk for intensive care, prolonged hospitalization or the development of respiratory failure [34]. Examination of hepatitis A virus (HAV) RNA from sera by real-time PCR resulted in higher initial viral load in patients with severe outcomes such as fulminant hepatitis and severe acute hepatitis than in patients with less severe infection [35]. This association has also been seen with other respiratory viruses such as influenza B or rhinovirus infections [36]. Viral load in patients infected with the pandemic type A influenza virus H1N1 (2009) who suffered pneumonia was higher than in patients with milder disease (those with bronchitis or upper respiratory tract infection), suggesting that viral load is also of important predictive value in influenza infection [37]. Other instances of a connection between viral load and disease progression have been reviewed [2–4].

Thus, viral load is emerging as a relevant viral parameter to predict the progression of COVID-19 and can be considered as a prognostic biomarker, together with other recognized risk factors for severity, in the management of COVID-19 patients.
